# Complete Immunophenotypic Reversal of Chronic Lymphocytic Leukaemia With High Dose Parenteral Methylcobalamin: A Case Report and Brief Review of Cobalamin in Cancer

**DOI:** 10.1002/cnr2.70106

**Published:** 2025-05-08

**Authors:** Carmen Wheatley

**Affiliations:** ^1^ Orthomolecular Oncology and Medicine, UK Reg. Charity 1078066, Oxford, UK; ^2^ St Catherine's College, University of Oxford Oxford UK

**Keywords:** cancer, chronic lymphocytic leukaemia, cobalamins, immunophenotypic reversal, nitric oxide, vitamin B12

## Abstract

**Background:**

Supposed ‘spontaneous’ remissions in chronic lymphocytic leukaemia (CLL) are extremely rare. By the most stringent immunophenotypic criteria, there are only seven cases to date of unexplained, immune system effected cures. A historic review of this phenomenon is presented as context for this eighth case of CLL immunophenotypic reversal.

**Case:**

A 59‐year‐old, molecular biologist, stage I CLL, whose diagnosis and recovery were both thoroughly documented, not content to watch and wait, chose to treat himself, after individual tumour susceptibility testing, with evidence based, biological response modifiers, which initially seemed to keep his CLL stable. This included 1 mg of hydroxocobalamin injected i.m. daily. However, after some years his lymphocytosis began slowly to drift upwards. At that point, he was persuaded to change his injection protocol to methylcobalamin, at 50 mg i.m. a day, a dose whose clinical safety is sufficiently well‐established, and a form of cobalamin that the research literature shows has anticancer actions.

**Conclusion:**

This change in cobalamin form and dose proved a critical turning point. Complete disappearance of the lymphocytosis also coincided with a severe infection and an even further temporary increase of the parenteral methylcobalamin dose, both catalytic factors. In the 4th and 5th years following this, the patient's repeated immunophenotyping showed no clonal disease present. A brief review of the field of cobalamin in cancer research and treatment is given, with discussion of the various mechanisms by which cobalamins may impact on cancer/CLL. Historic analysis reveals that cyanocobalamin is generally cancer promotional, whereas hydroxocobalamin, methylcobalamin and adenosylcobalamin are cancer protective and cytotoxic. It is hypothesised that the actions of cobalamin in cancer aetiology and oncogenesis/progression are intertwined with those of nitric oxide, which tumours regulate to dupe the immune system to their presence, by causing a functional cobalamin deficiency in the host.

## 1 Introduction

Chronic lymphocytic leukaemia (CLL), a B lymphocyte neoplasm, and the most commonly occurring of the leukaemias, accounts for under 1% of all cancer deaths worldwide [1]. CLL is a relatively benign haematological malignancy. Survivals vary between CLL mutation types [2], but, typically, can range between 8–10 years and up to 20–25 years. Chronic Lymphocytic  Leukemia Treatment (PDQ®)–Health Professional Version ‐ National Cancer Institute. www.cancer.gov. 2020‐10‐09), with improved outcomes from current therapy in those who do not have chromosome 17 or 11 abnormalities, and who have an unmutated immunoglobulin heavy‐chain, IgVH [3, 4]. While the newer CD20 monoclonals in combination with the Bruton tyrosine kinase inhibitor, ibrutinib, the B‐cell lymphoma 2 inhibitor, venetoclax, and idelalisib, a phosphoinositide 3‐kinase δ inhibitor, promise a potential cure, this has yet to be established as reality. Furthermore, even if the current standard FCR therapy combination of Fludarabine, Cyclophosphamide and the CD20 monoclonal, Rituximab offers longterm disease free survivals for approximately 30%–40% of patients with good cytogenetics at diagnosis, this treatment still involves the not inconsiderable rigours of chemotherapy and potential serious adverse side effects of Rituximab. It also leaves CLL patients with poor cytogenetics less than optimally served.

The indolence of CLL progression has led many clinicians to adopt a watch and wait approach to early CLL, in particular, because there is no agreed standard treatment at this stage, or sufficient evidence that early intervention can prevent eventual progression [5]. While, as stated, a variety of treatments are possible in the later stages of CLL, there has to date been no truly curative therapy, with the possible exception of immunotherapy with individual genetic manipulation of T cells in a handful of experimental cases [6, 7].

However, the CLL literature contains accounts of rare, so‐called spontaneous remission [8]; estimated to have an incidence of 1% [9], but in reality, more like 0.6% [8], with some 45 cases reported in a CLL literature span of 70 years [9, 10, 11, 12, 13, 14, 15, 16, 17, 18, 19, 20, 21, 22, 23, 24, 25, 26, 27, 28, 29, 30, 31, 32, 33, 34, 35, 36, 37]. Such remissions are understood to be mediated via the immune system [38], by triggers that remain somewhat mysterious, though there are clues: some of the cases of spontaneous remission have followed on either from vaccination [15, 16, 39], or after viral infections [14, 17, 26, 30], and sometimes even with occurrence of other haematological neoplasms [13, 18, 27, 32], thrombocytopenia [35] or bone marrow necrosis [19]. An independent review of ‘spontaneous’ remissions in CLL actually identified 92 case abstracts after an extensive PubMed search, but then narrowed the real incidence of remission down, first to 44 cases, based on stringent criteria, and ultimately, to only 21 cases which actually met immunophenotyping criteria, details of sufficient duration/stability of remissions, and lack of any standard treatments as an obvious cause of remission [35]. Further, of these 21 cases only 7 showed complete reversion to normal after repeat immunophenotyping, whilst the remaining 14 cases showed a persistent CLL clone present in spite of an apparent normal baseline lymphocyte count [35].

Such rare incidences of complete CLL reversal provide a context for this case study, which first reports the complete immunophenotypic reversal of early stage CLL with a novel, safe, immunotherapeutic approach, using high dose parenteral methylcobalamin/MeCbl, one of the two active coenzyme forms of vitamin B12, known to have profound effects on the immune system, as well as in cancer: (for extensive reviews cf. Wheatley, 2025, The Effects of Cobalamin on the Immune Response; Wheatley, 2025, Cobalamin, Nitric Oxide and Cancer. *In Tech*. In preparation).

## 2 CLL Case History

The patient, a male, molecular biologist, and ex MRC/MIT researcher, was diagnosed with CLL in November 2007, at 59 years of age. The initial diagnosis coincided with the commencement of a painful bilateral hip arthritis. Investigation of the arthritis led to the chance discovery of early CLL, for which the patient was asymptomatic. A mild B lymphocytosis with moderate numbers of smear cells was noted, and (the patient being a scientist with OCD), this was confirmed repeatedly by different laboratories (Doctors Laboratory, London; the Cromwell Hospital, London; the Princess Elizabeth Hospital, Guernsey; Southampton Hospital; the Royal Marsden Cancer Hospital, London; the John Radcliffe Hospital, Oxford) over the course of 2 months, with the B lymphocytosis values ranging through: 9.08, 10.6, 11.38, 11.6, 12.2, 10.1. Review of past medical records revealed an already mildly elevated lymphocyte count of 7 in late May 2006, and a possible baseline of 4.3 in early November 2004. On 7 December 2007, tests at the Royal Marsden showed a normal immunoglobulin profile, with IgG just over the top end of its normal range at 13.3 G/L, CRP < 2 mg/L, LDH 182.

In late November 2007, a consultant haematologist at the John Radcliffe Hospital, Oxford, confirmed the patient's diagnosis of Binet stage A CLL, with no palpable lymphadenopathy or hepatosplenomegaly, normal haemoglobin of 14.6, and normal platelet count at 253. The WBC was then 18.9, and lymphocytosis 11.38. The Oxford immunophenotyping report showed a gated lymph population at 49% of normal: positive for CD19, CD5, Lambda, CD23, CD20, IgM; negative for FMC7, CD79B, and CD38. Molecular cytogenetic analysis found no evidence of p53 deletion in 200 cells examined.

Subsequent immunophenotyping (from a second opinion consultation at the Royal Marsden hospital, London, in the first week of December 2007), also confirmatory for CLL, revealed ZAP‐70 as negative (at 20% cut‐off), and a positive 19% CD38 (at 7% cut‐off). Clonal B cells were seen to represent 40% of total peripheral blood cells with phenotype. These were positive for CD5, CD19, CD20, CD22, CD23, and FMC7, weakly positive for CD79B, and clonal for lambda. A CLL score of 3/5 was assigned to the patient at the Royal Marsden.

Southampton hospital results again confirmed this lymphocyte profile, noting lymphocytes were also negative for CD10, FMC7, CD73b, and CD18. Molecular cytogenetics at the Royal Marsden showed no p53 deletion, and DNA studies noted hypermutated immunoglobulin genes, indicative of a good prognosis. LDH in mid‐December 2007 was 374. The beta 2 microglobulin/B2Mcg (tested late February 2008) was just at the top end of the normal range at 2.3 mg/L. FISH (fluorescent in situ hybridisation) analysis revealed 10% 17p deletion, incomplete 13q deletion, and 11q negative status. The Marsden FISH molecular cytogenetic analysis was unable to study the mutational status of the IGH genes due to lack of amplification of the VDJH rearrangements (FR1, FR2, and FR3), which was attributed to a high rate of somatic hypermutation in the IGH genes, a somewhat unusual finding in CLL, more typically seen in (post) germinal centre‐B‐cell lymphoproliferations. Analysis of DJH and KDE genes showed clonal rearrangements, consistent with the presence of a clonal B cell population.

## 3 Additional Non‐Standard Molecular Diagnostic Tests

The patient's distinguished research background as a molecular biologist, (ex‐Fellow of Trinity, Cambridge/MRC/MIT), and his dissatisfaction with the lack of early treatment for CLL, also led him to pursue non‐standard experimental options both in diagnosis and early treatment. In addition to the standard tests for CLL just described, whole blood samples were sent to Dr. Ioannis Papasotiriou's Cancer Genetic Research Centre laboratory in Greece.

The aim of the GCRC lab tests is not just to genetically profile individual cancers, but to propose epigenetic solutions to particular genetic predispositions or targets, by testing a comprehensive range of both drugs and biological response modifiers against a patient's tumour cells.

Thus at the GCRC lab the patient's malignant clonal B lymphocytes were isolated after centrifugation, using Oncoquick with a membrane that isolates the clonal cells by means of positive selection with monoclonal antibodies/Mabs against CD19, CD20, CD38 and negative selection using anti‐CD45 Mabs. The resulting cell populations were then cultured and first tested against a range of chemotherapeutic, immunotherapeutic and biological response modifiers, for cytotoxicity potential: (for full data details and Materials and Methods used, cf. Supporting Information). Second, an extensive genetic profile of the tumour cells was compiled, looking at the status of genes that might impact in any way on tumour progression. This genetic profile included screening for genes promotional for tumour cell angiogenesis and growth factors (vascular endothelial growth factor [VEGF], fibroblast growth factor [FGF], platelet derived growth factor [PDGF]; transforming growth factor beta [TGFβ], epidermal growth factor [EGF], insulin growth factor [IGF]; COX2, 5‐LOX); matrix metalloproteinases; transcription factors (NFκB, IκB‐a,d,e), including cell cycle regulators (p53, p16, p27—a CDK inhibitor—, p180); DNA synthesis (ribonucleotide reductase [RR]); DNA methylation (DNA methyltransferase‐1 [DNAMT‐1], DNA demethylase, O6‐methylguanine‐DNA transferase); histone deacetylase activityś; genes involved in resistance to certain chemotherapeutic drugs (CES1 and 2, DPD, UP, NP, TP, Gamma GC); rapid cell cycle related genes (TS, DHFR, SHMT, and GARFT); CD20; CypB1; Her/neu2 and Her 1 related genes (c‐erb‐B2 and c‐erb‐B1) and genes associated with a resistant or aggressive tumour phenotype (Bcr‐abl and h‐TERT).

## 4 Results of the Clonal B Cell Gene Profiling and Tumour Drug Sensitivity Testing

**FIGURE 1:**
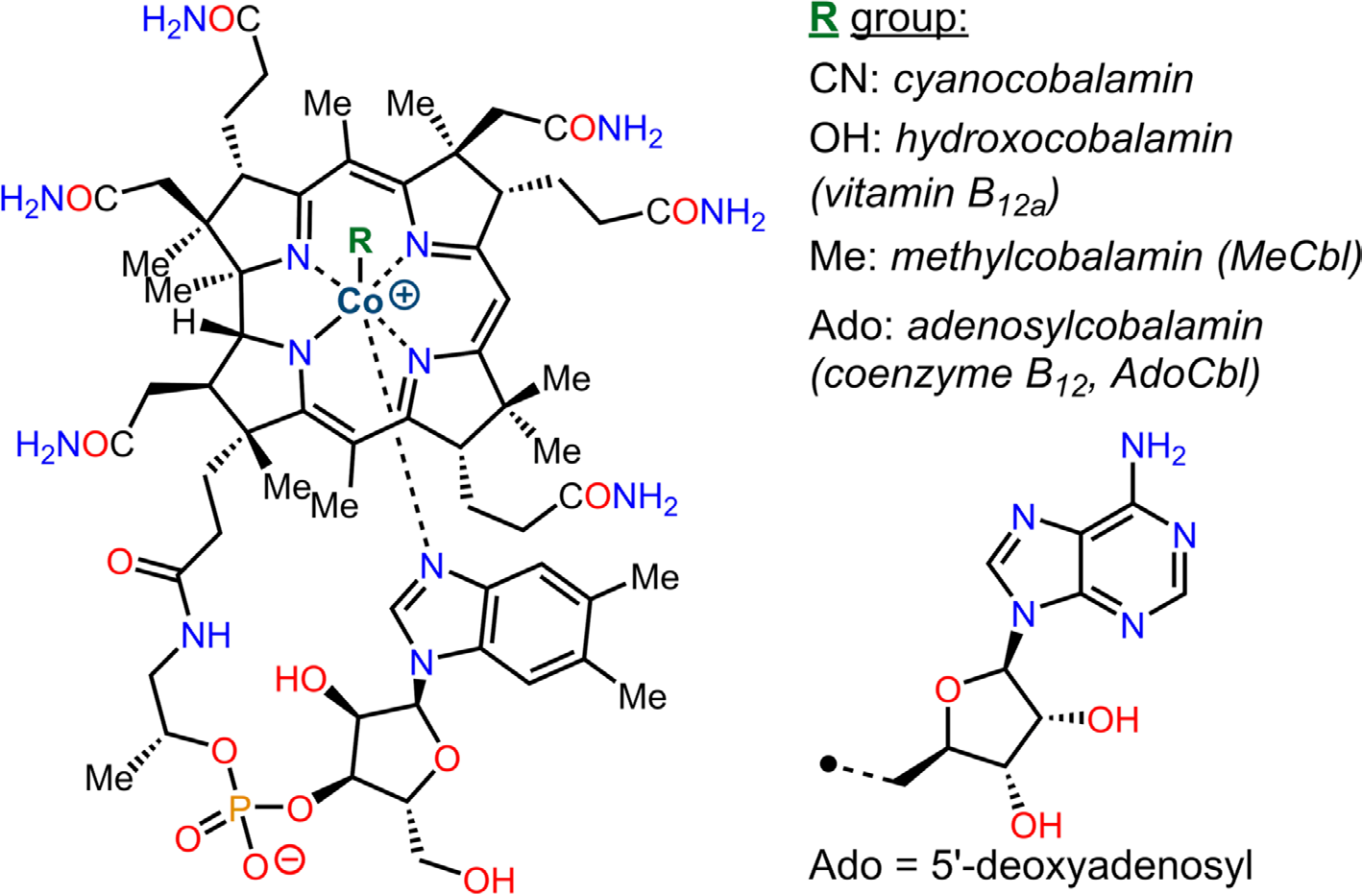
The structure of Cobalamin.

MDR1, the multi drug resistance 1 gene, was 65% over‐expressed as compared to control, suggesting some resistant clonal B cell populations even at this early stage of the patient's CLL. Dr. Ioannis therefore recommended the patient consider the use of either of two drugs known to downregulate MDR1, namely, the calcium channel blocker, verapamil [40], or imidazole compounds, specifically, the antifungal, ketoconazole [41]. However, in the event, the patient, at this author's instigation, had already planned treatment with parenteral cobalamin, which has multiple anticancer actions. In vitro data also demonstrates that cobalamin significantly effects downregulation of MDR1 [42], through phospholipase D activation [43]. Moreover, it is notable that the structure of cobalamin incorporates a lower axial dimethylbenzimidazole ligand, thus, a naturally occurring imidazole (making ketoconazole redundant): Figure 1. MDR1 itself has, coincidentally, been shown to be the native cobalamin export pump [44].

Other key data of the patient's gene profile showed activity of glutathione S‐transferase (GST) and gamma glutamylcysteine synthase in the low limits, both of which corresponded to lack of tumour cell resistance to platinum compounds, albeit chemotherapy is not a standard option at such a stage of CLL. Carboxylesterases CES1 and CES2 in the normal range should correspond to zero resistance of the tumour cells to camptothecin compounds, topoisomerase I inhibitors. There was increased activity of laminin and matrix metalloproteinases (MMP), corresponding to the CLL tumour cells' increased invasive ability. However, some research evidence indicates that cobalamin is an MMP inhibitor. There was no tumour cell sensitivity to taxanes (paclitaxel, docetaxel), but significant sensitivity to alkaloids, in particular, to vinca alkaloids such as vincristine.

Minimal tumour cell sensitivity was observed for the antifungal 5‐fluorocytosine (5FC), the chemotherapeutic agents, fluorouracil (5‐FU), tegafur‐uracil (UFT), floxuridine (FUdR), capecitabine, raltitrexed, pemetrexed and gemcitabine, but there was significant sensitivity to methotrexate and fludarabine. Increased tumour cell sensitivity was also seen with alkylating agents, in particular, to bendamustin; to inhibitors of topoisomerase IIa and IIb, in particular, to mitoxantrone. But, paradoxically, given the lack of resistance to camptothecin compounds indicated by CES1/CES2 levels, there was no tumour cell sensitivity to such inhibitors of topoisomerase I. Nor to dacarbazine (more commonly used for melanoma and Hodgkin's lymphoma). Taurolidin, an antimicrobial with potential repurpose for cancer [45], at two different doses, also failed to induce apoptosis.

Further of note in the patient's tumour susceptibility screen were high levels of transforming growth factor beta/TGFβ (30% over control); of the transcription factor, NFκB (35% over control) together with inhibition of IκB (a,d,e) expression; and somewhat raised epidermal growth factor receptor/EGFr (< 5%). Moreover, all the growth factors tested for were also over‐expressed. Indeed, the high neo‐angeogenesis potential of the tumour cells was highlighted by the over‐expression of VEGF‐receptor 40% over control. Whereas there was no over expression of somatostatin receptors (SS‐r); 5‐lipoxygenase (5LOX); oestrogen receptor; progesterone receptor; dehydrotestosterone receptor; cycloxygenase‐2 (COX2); the proto‐oncogenes, and receptor tyrosine kinases, EGF2 receptor/c‐erb‐B2, EGF1 receptor/c‐erb‐B1. Finally, it was observed that **t**he three key heat shock proteins/Hsp tested for were all significantly inhibited: with Hsp27 downregulation at 65% under control, Hsp90 at 20% below control, and Hsp72 15% below control. (cf. Supporting Information). This was an interesting finding, as high levels of the housekeeping proteins and chaperones, heat shock proteins play tumour cytoprotective roles in respect of growth and prevention of cell death in leukaemias and lymphomas [46].

This information added to the patient's rationale for his choice of treatment with cobalamin, since cobalamins are key regulators of heat shock proteins [47].

## 5 Results of the Clonal B Cell Gene Biological Response Modifier Sensitivity Testing

Thirty nine cultures in foetal calf serum were made from the patient's isolated clonal B lymphocytes, and one of the following biological response modifiers was added to each well: hydrogen peroxide (H_2_O_2_), ascorbic acid [48], carnivora (extract of *Dionaea muscipula* ), misteltoe, quercetin, indole‐3‐carbinol, c‐statin (an extract of *Convolvulus arvensis* leaves), ukrain (an alkaloid extract from the plant *Chelidonium majus* ), polyMVA (a cocktail of alpha lipoic acid, vitamins, minerals and amino acids), co‐enzyme Q10, Essiac tea (a blend of burdock root, *Arctium lappa* , Indian rhubarb, *Rheum palmatum* , sheep sorrel, *Rumex acetosella* , and the inner bark of slippery elm, *Ulmus fulva* or *Ulmus rubra* ), modified citrus pectin, IP6 (inositol hexaphosphate [49]), pancreatic enzymes, salvestrol (a phytonutrient extract which can interfere with tumour cell detoxifying enzyme CYP450 and CYP1B1 upregulation [50]), Cat's Claw, (*Uncaria Tomentosa*, rich in the pentacyclic oxindole alkaloid, mitraphylline), carctol (a mix of Indian herbs, *Hemidesmus indicus*, *Tribulus terrestris* , *Piper cubeba* , *Ammani vesicatoria*, *Lepidium sativum* , *Blepharis edulis*, *Smilax china* *and Rheum australe*), noni juice ( *Morinda citrifolia* ) [51], acetogenins from the Annonaceae plant family [52], caesium chloride [53], reolysin (a proprietary oncolytic isolate of the unmodified human rheovirus [54]), amygdalin‐B17, artesunate [55] maitake mushroom extract [56], the carotenoid, lycopene [57], curcumin [58, 59], green tea polyphenol extract as epigallocatechin‐3‐gallate (EGCG) [38, 60, 61, 62, 63, 64], the sleep hormone, melatonin [65, 66], ellagic acid [67], l‐methionine, N‐acetyl‐cysteine [68], niacin [69], l‐carnitine, vitamin E as tocopherols, superoxide dismutase (SOD) [70], propolis [71], selenium [72], *aloe vera* , interferon 2 alpha (IFNα) [73]. In addition, in a separate assay, a combination of vitamin D3 with ascorbic acid at two different ratios was also tested against the tumour cells. Samples of the 39 cultures were harvested every 24 h for 6 days and the following assays performed. All biological response modifiers were tested for their tumour cell apoptosis inducing effects, using caspases 3, 9 and cytochrome c as primary indicators of apoptosis. Additionally, in the cultures containing carnivora, ukrain or mistletoe, the activity of tyrosine kinase, and cytokine production, as a result of expression of high CLL cellular expression of EGFr and insulin growth factor receptor (IGFr), were both measured. In the culture containing quercetin, inhibition of EGF and IGF growth factors was analysed. In the culture containing indole‐3‐carbinol, inhibition of the growth factors VEGF, FGF, platelet derived growth factor (PDGF), was analysed. In the culture treated with H_2_O_2_, cell viability after 4 days of treatment was analysed. In the cultures treated with either ascorbic acid, or PolyMVA, the catalytic activity of glutathione (GSH) and its oxidised form GSSG, together with induction of cytochrome c, a mitochondrial marker of apoptosis [74], were analysed. Finally, in the culture treated with artesunate both levels of GSH and GSSG, induction of mitochondrial cytochrome c, and inhibition of the growth factors VEGF, FGF and PDGF were analysed.

## 6 Key Findings From the Biological Response Modifier Testing of the Patient's CLL Cells

Only a very few of the many biological response modifiers tested proved to have any significant anti‐tumour actions in the patient's CLL. These agents comprised: ascorbic acid and c‐statin, which both increased caspases, in particular, caspases 3 and 9, by 55%; quercetin, which inhibited EGF by 60% and IGF by 45%; salvestrol, which upregulated caspase activity, in particular, caspases 3 and 5, and also expression of cytochrome c, by 65%, while decreasing the viability of the culture by 45%; artesunate, which increased expression of cytochrome c by 45%, whilst also decreasing intracellular free radicals, notably the GSSG/GSH ratio, with a corollary inhibition of VEGF by 35%, FGF by 30% and PDGF by 25%. The only other significant finding was in the propolis treated culture, which showed both a 40% increased caspase activity, in particular caspases 3 and 9, and a 40% increased cytochrome c expression.

## 7 The Initial Experimental CLL Treatment Protocol

Since it was not standard at the time to treat early CLL with chemotherapy, or the newer immunotherapeutic anti CLL drugs, the patient nonetheless now had a rational basis for a non‐drug based, tumour personalised, experimental early treatment protocol. For the next decade the patient's early CLL treatment protocol, which it was hoped might prevent progression of the CLL, consisted of the following: oral ascorbic acid, 8–10 g divided into 3 daily doses; 600 mg alpha lipoic acid to recycle the vitamin C [75, 76], quercetin, 2 × 167 mg capsules daily; salvestrols, 6 a day. The patient chose not to take anti‐angiogenic c‐statin, (an extract of convolvulus), nor the anti‐malarial drug, artesunate, the latter on grounds of its potential for adverse haematological effects. Moreover, while the green tea extract EGCG had no significant effect in the lab culture tests, the patient decided to nonetheless incorporate this into his experimental protocol, on grounds of the known general beneficial actions of EGCG in CLL [60, 61, 62, 63, 64, 77]. The daily dose of EGCG taken was somewhat above the range of that in a small Mayo Clinic series case report, which saw objective clinical responses in four out of four patients (three with CLL, one with follicular lymphoma), treated either with 2 × 315 mg capsules of EGCG, or multiple green tea drinks daily, for 12–15 months and ongoing [62]. Since the CLL literature also shows some synergy between EGCG and curcumin [58], the patient also incorporated curcumin into the protocol (4 × 900 mg a day).

As mentioned earlier, the patient decided against either verapamil or ketoconazole as MDR1 inhibitors, and chose instead to use parenteral cobalamin as an MDR1 inhibitor, initially, and for nearly a decade, as daily i.m. injections of hydroxycobalamin (HOCbl), at the relatively low dose of 1 mg. This was done post testing at the GCRC lab. His diet, which always contained good quantities of fresh fruit and vegetables, olive oil and healthy protein, such as fish, poultry, eggs, cheese, remained largely unmodified, except for the addition of daily green juices for a time.

## 8 Progress

**FIGURE 2:**
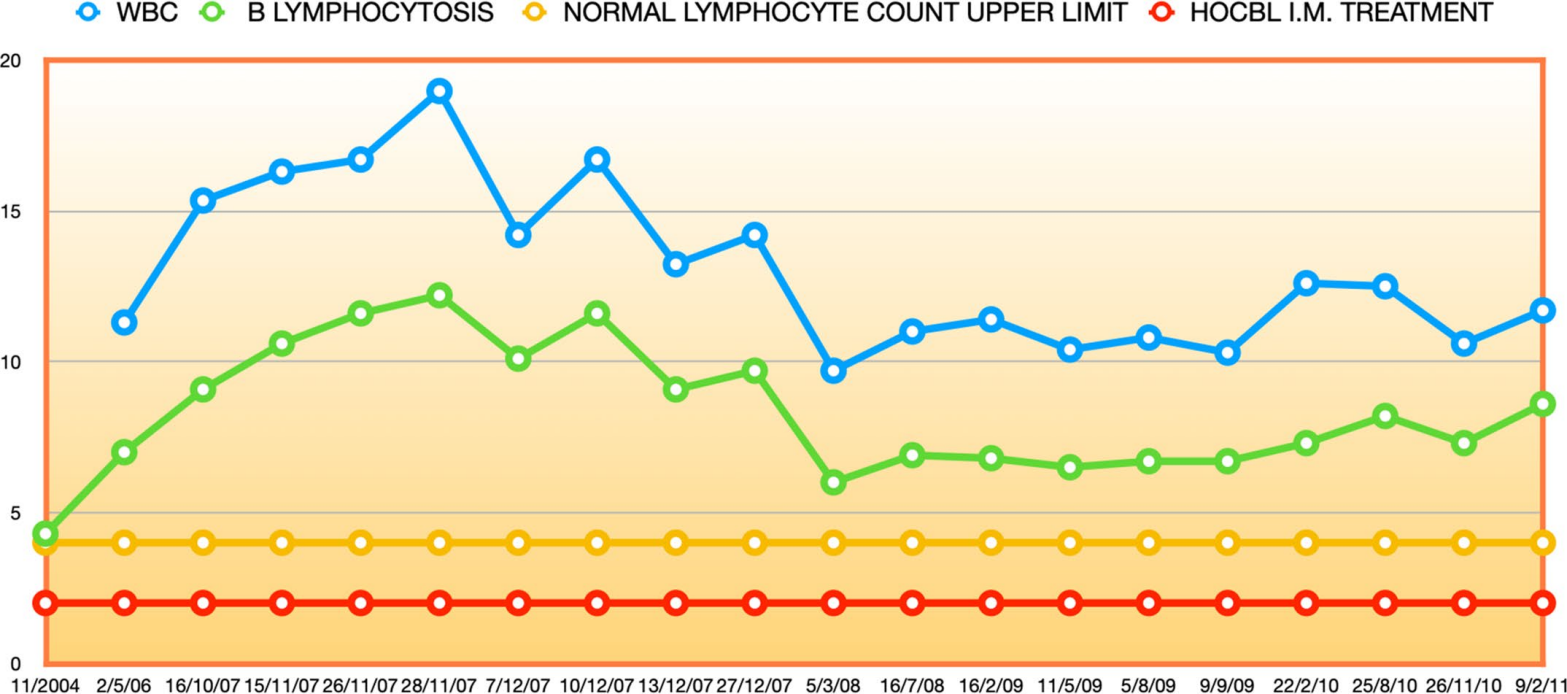
CLL monoclonal B lymphocytosis, 2004–2011. From 2008: Under low dose i.m. Hydroxocobalamin treatment.

Five years passed. The patient had regular blood tests to monitor the lymphocytosis, which remained largely stable but clearly present: Figure 2.

The patient ascribed his relative stability to the experimental protocol. However, given the well‐known slow progression of CLL, and initial good prognostic markers, it could be argued that the protocol may have been irrelevant and had no impact on his status.

**FIGURE 3:**
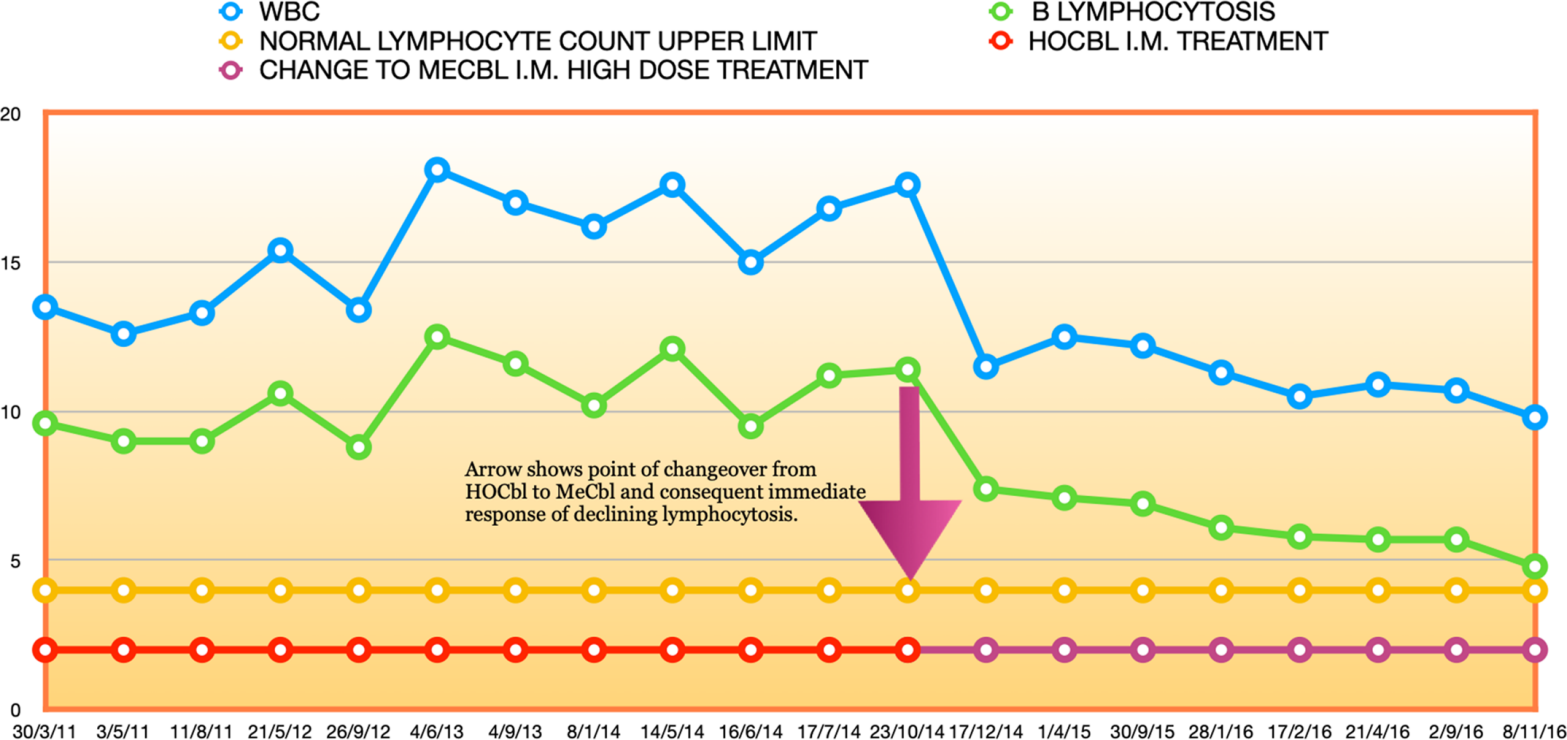
CLL monoclonal B lymphocytosis, 2011–2016. A significant response and descent to near normal values during the switch from relatively low high dose 1 mg i.m. Hydroxocobalamin daily to high dose 50 mg i.m. Methylcobalamin daily treatment.

Indeed, as if to confirm this, from May 2012 to October 2014 there was a noticeable upward drift of the lymphocytosis, towards levels nearer to those at the point of diagnosis: compare Figures 2 and 3, up to the 23/10/2014 point.

What happened next, however, may be much harder to dismiss.

**FIGURE 4:**
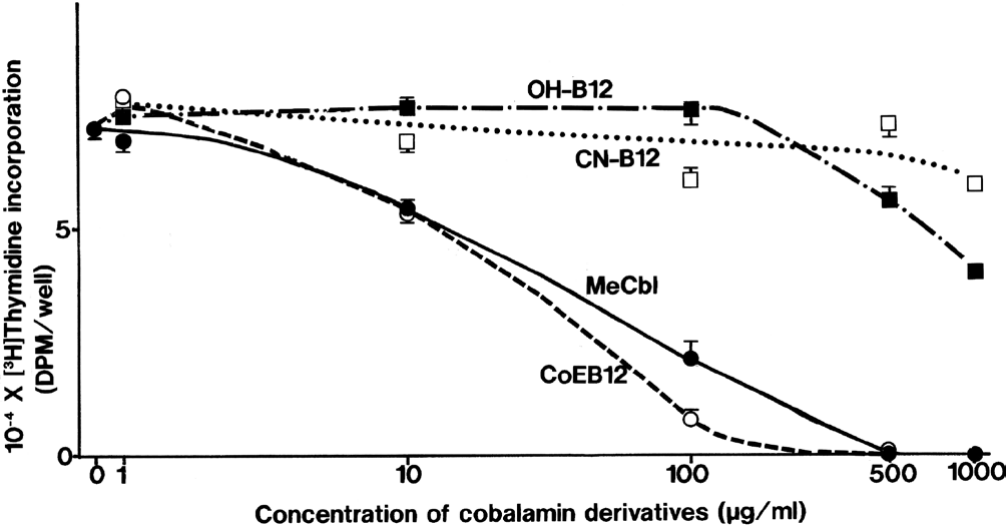
Effects of coenzyme and ‘inactive’ cobalamin forms on the proliferation of SC‐3 cells. SC‐3 cells were plated onto a 96‐well plate (3 × 10^3^ cells/well) containing 0.15 mL of MEM with 2% DCC‐treated FBS. On the following day (Day 0), the medium was changed to 0.15 mL of MEM containing %2 DCC‐treated FBS and 10° M testosterone in the absence or presence of various concentrations of cobalamin derivatives. On Day 3, the cells were pulsed with [3H]thymidine (0.15 mcg Ci/0.15 mL per well) for 2 h at 37°C, and the radioactivity incorporated into the cells was measured. CN‐B12, cyanocobalamin; CoB12, adenosylcobalamin; MeCbl, methylcobalamin; OHB12, hydroxocobalamin. Points, means of four determinations; bars, SE. The other three separate trials also gave similar results.

The patient was made aware by the author of this paper that he had not tapped the full potential anti‐cancer action of cobalamin, which, critically, according to my extensive analysis of all the cobalamin cancer research literature (cf. Section 9 and summary table there), appears to be strictly dependent both on dose, and on cobalamin form. This thesis is neatly encapsulated in the work of Dr. Nishizawa and his group in Japan over two decades ago. It is clear from all Dr. Nishizawa's research that the active coenzyme forms of cobalamin, methylcobalamin (MeCbl) and adenosylcobalamin (AdoCbl; CoeB12), are very significantly more cytotoxic than HOCbl, or CNCbl; that MeCbl and, in particular, AdoCbl, have a very clear tumour cytotoxicity dose response, which increases significantly as the dose is raised. This is illustrated well in one of his group's various comparable studies [78] (results reproduced here by kind permission of Dr. Nishizawa): Figure 4.

Dr. Nishizawa's in vitro study outcome of the diverse effects of cobalamins on the growth and viability of Sc3 androgen dependent mouse mammary tumour cells [78] shows almost no effect of CNCbl, a negligible effect of HOCbl, at all but astronomic doses, certainly not comparable to the relatively trivial high dose of 1 mg HOCbl that the patient had been injecting daily for years, without any obvious anticancer effect. So, based on this insight, and the weight of all the cobalamin and cancer lab research evidence, the patient stopped injecting HOCbl and changed instead to MeCbl, at a significantly much higher dose of 50 mg i.m. daily. (For the research based rationale of this dose cf. Section 9). Safety was not a concern since there is an extensive and excellent pharmacological safety profile for parenteral cobalamin deployed at significantly much higher doses, —HOCbl being used for nearly 70 years in the ICU, at 5 g IV, as the anti‐cyanide antidote in France, Italy, China and elsewhere, and in later years, in the United States. Moreover, the patient's daily 50 mg parenteral dose of MeCbl had already been clinically safety tested thrice, in Japan: first, in a 6 month trial, where patients with advanced chronic MS injected 60 mg i.m. daily, and second, in a pre‐clinical study where ALS patients injected 25 mg i.m daily for 14 days, and then, again, in a clinical trial where ALS patients injected either 25 or 50 mg i.m. twice weekly [79, 80, 81]. The trials saw some good outcomes, without adverse effects, and thus the ALS clinical trials of the 50 mg dose have continued to the present, and are now in Phase III clinical trials [82].

**FIGURE 5:**
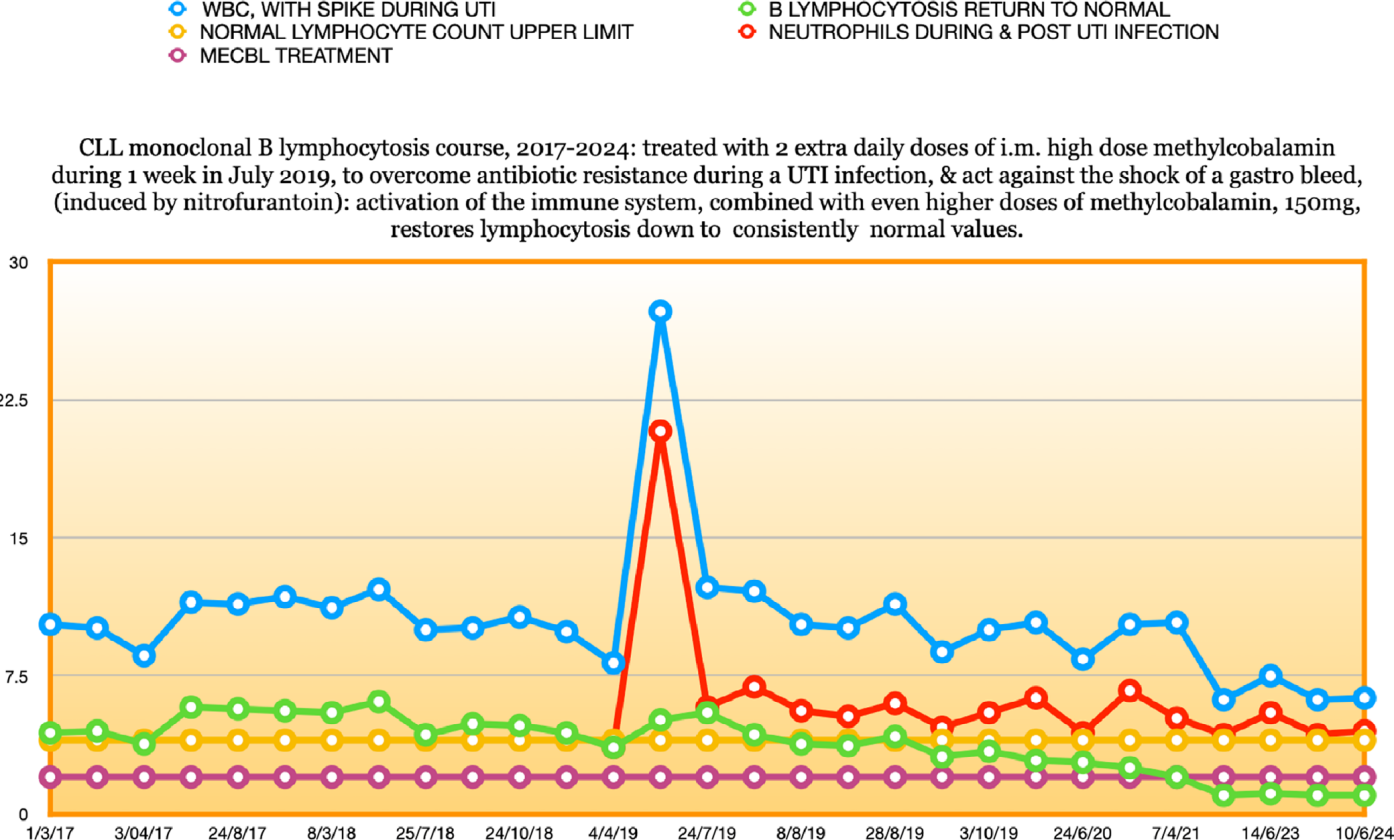
Lymphocytosis vanishes in 2019 after a severe UTI and with increased MeCbl dose. Repeat immunophenotyping in 2023/2024 shows no clonal disease present.

As Figures 3 and 5 show, within a few weeks of beginning this new daily high dose i.m. MeCbl protocol, in November 2014, the patient's lymphocytosis began a descent towards the normal range.

From early April 2017 to April 2019, while the regular high dose i.m. MeCbl treatment was continued, the lymphocyte count repeatedly touched the normal range, or hovered just above it (Figure 3 and first section of Figure 5), and then, something even more interesting occurred.

At the beginning of July 2019, the patient contracted a serious UTI infection. This proved resistant to the first antibiotic prescribed, nitrofurantoin, which antibiotic actually made the patient's physical condition even worse, and, additionally, triggered psychosis. On switching to a new antibiotic, (Trimethoprim), it was decided, since the patient was extremely unwell, and there was no safety concern, to triple the dose of i.m. MeCbl each day, to 150 mg, in order to prevent further antibiotic resistance. In the event, it was extremely fortunate that this was done, since, unbeknownst to him, but observed by his caregiver, the patient, just at the point of antibiotic switchover, suffered a considerable gastric bleed, at home. Yet, strikingly, although he looked like Banquo's ghost, and his normally high blood pressure dropped significantly down to a low normal, the BP stabilised there and the patient did not go into shock, most likely due to the known anti‐shock actions of very high‐dose parenteral cobalamin, as observed first in a Russian study in dogs [83]. The patient resisted going into hospital for some days, eventually going in for a gastroscopy, and some observation, after oedema was observed—but no ulcer—at the head of the duodenum.

One month later, the B lymphocyte count was under the normal upper limit base line, and has remained there consistently to this day in March 2025: Figure 5. The patient's blood has remained normal and completely free of his B lymphocytosis for more than 5 years now. The patient, now 76, is at present alive and well, without any symptoms of CLL. A repeat CLL immunophenotyping in early 2024, after the first test in spring 2023, shows that there is no clonal disease present. In effect, the patient has been cured. The acute UTI infection, combined with the transient escalation of MeCbl dosing during the infection appeared to be the turning point. So that, in the context of the CLL MeCbl immune modulating therapy, paradoxically, the UTI infection would appear to have acted on the CLL as a form of ‘Coley's Toxins’ provoked immunotherapy.

Perhaps one should not discount also a possible synergy in anti tumour action of the MeCbl and an activated immune response with the non‐classical antifolate actions of Trimethiprim (C_14_H_18_N_4_O_3_):5‐(3,4,5‐Trimethoxybenzyl)pyrimidine‐2,4‐diamine).

Moreover, the cobalamin cancer lab research literature shows there can be such a cytotoxic synergy between the classical anticancer antifolate, methotrexate and MeCbl. A 1978 Russian study in six different strains of mice, tested methotrexate and MeCbl both alone, and in combination, as treatments for a range of tumours (breast, gut, cervix, adenosarcoma and sarcoma), and, paradoxically, found no effect of either agent alone (indeed, even a slight promotional effect of MeCbl), but a very definitive anti‐cancer effect when both agents were combined [84].

## 9 Discussion

The more than half a century of research on cobalamin in cancer treatment, largely in vitro, in animal studies, including in dogs [85, 86], but comprising also a very few clinical case studies and trials, (including an important 10 year clinical trial of parenteral cyanocobalamin [CNCbl] for children and infants with terminal neuroblastoma, who had failed surgery and radiation, at the Great Ormond Street Hospital for children in the 1950s era before chemotherapy [87]), amounts to some 191 diverse, individual in vitro, in vivo and a very few clinical studies, now also including this and six other case series [88, 89, 90, 91, 92].

Cobalamin is a central regulator of the immune response (reviewed in [93, 94, 95]), critical for immune cell and platelet, red cell production. Therefore it is not surprising that cobalamin deficiency and pernicious anaemia are epidemiologically associated with increased cancer incidence and susceptibility, in particular, of gastric cancer and haematological malignancies [96], including CLL [97]. Yet, it is widely believed by many doctors, and researchers, that if you have cancer, taking or injecting cobalamin is a dangerous and cancer promotional thing to do. Indeed, to this day there is a small research literature based on this dogma bent on devising ways of blocking cobalamin entry into cancer cells [98]. However, this dogma is completely misguided, and a vital discovery, after my review of the entire 70 year cobalamin and cancer field, needs to be taken on board: as it turns out, most forms of cobalamin/B12 are cancer protective/cytotoxic: but B12 *as cyanocobalamin* can be cancer promotional. Yet, the misapprehension and dogma persist due lack of an overview of the field and to poor studies. For example: a recent epidemiological study correlated higher dose ingestion of vitamins B6 and B12 with a 30% to 40% increased incidence of lung cancer in smoking, or ex‐smoker men, though notably *not* in women, or in non‐smokers [99]. However, astonishingly, no record was made of *which* cobalamin form the VITAL study participants had ingested. This is crucial missing information.

Review of the field of cobalamin/vitamin B12 and cancer offers clarification. Table 1 (which is just a summary table of a highly detailed analytical table in a forthcoming extensive Review of the entire 74 year field of cobalamin and cancer studies [100]), shows a basic analysis of 192 international studies and individual experiments within the studies, from 1949 onwards, of the effects of different cobalamins in a great variety of cancer cell lines/models, in vitro, in vivo, and in a few clinical trials: Table 1.

**TABLE 1:** Effects of cobalamins in diverse tumour models to date.

	Promotional	No effect	Anti‐cancer
CBL forms × number of studies			
Cyanocobalamin × 69	35 = 50.72% during induction, in the presence of carcinogens, or for existing tumours.	12 = 17.64%	22 = 32.35%. In neuroblastoma, & melanoma.
Hydroxocobalamin × 36	N/A	4 = 11.11%	32 = 88.88%
Methylcobalamin × 23	1 = 4.34%	1 = 4.34%	23 = 91.30%
Adenosylcobalamin × 14	N/A	3 = 21.42%	11 = 78.57%
Nitrosylcobalamin × 58 [46 cell lines, 7 rodent, 5 dog studies].	N/A	N/A	58 = 100%

What emerges from all the cobalamin and cancer research literature to date, as this summary table shows, is that not only do other forms of cobalamin not have the potentially significant cancer promotional effects of CNCbl: they are actually cancer protective, and, in supra‐physiological doses, are tumour cytotoxic, in particular the two active forms, MeCbl and AdoCbl. This conclusion is consistent with the known protective effects of cobalamin on genomic integrity and regulated methylation, as well as with the slowly decreasing risk for diverse cancers during treatment and recovery from cobalamin deficiency/pernicious anaemia.

Studies referred to by Brasky et al. as supportive of their questionable and indiscriminatory conclusion about B12 actually all used CNCbl. Related studies which point to total serum B12 as proof that high B12 levels drive cancer, are also misleading, as 80% of serum B12, on the second cobalamin carrier, haptocorrin, is *not* bio‐available, so that measuring total serum B12 is not helpful [101]. Yet, paradoxically, many studies show that serum B12 levels, mostly on haptocorrin, increase as cancers progress [102, 103]. This shunting of cobalamin by tumours into the circulation usually means that the cancer patient host is functionally cobalamin deficient, whilst the tumour has just the right amount of cobalamin it needs in order to replicate. It is this functional host cobalamin deficiency that drives cancer, not the fact that the tumour requires B12 to grow. By making the host functionally cobalamin deficient tumours are able to blind the immune system to their presence. Giving high doses of active forms of cobalamin can therefore undermine this tumour strategy, alerting the immune system to the tumour in its midst.

Until quite recently, the standard form of B12 in American supplements was CNCbl. The information from the VITAL cohort, recruited in 2000–2002, actually derived from the previous decade. It is a reasonable assumption that, even ongoing, what was ingested at the time was CNCbl (as MeCbl was *not* then widely available), and the Cbl routinely found in multivitamin supplements of the time—and even often today—was, almost invariably in the United States, CNCbl, essentially, an excretory form of cobalamin.

Linnell et al. long ago remarked on the significantly higher serum levels of both cyanide and CNCbl in smokers, their higher urine excretion of CNCbl. It was already known that high levels of cyanide deregulate B12 enzymatic pathways and deplete body B12 stores [104], thus creating a functional, or absolute cobalamin/B12 deficiency, with consequent deregulation of gene methylation and of mitochondrial energy production, both hallmarks of cancer.

CNCbl is also a known nitric oxide synthase inhibitor [105], whereas nitric oxide/NO* is cancer protective/tumour cytotoxic [106]. So here is another mechanism for CNCbl's cancer promotional effects. It happens that oestrogen also increases protective NO* production [107] and this may explain the better outcomes of women in VITAL. Notably absent from the VITAL study's references are two randomised, double blind clinical trials which show that, by contrast, hydroxocobalamin/HOCbl and MeCbl reverse precancerous squamous metaplasia in smokers' lungs [108, 109].

In line with the importance of the right cobalamin form for cancer treatment, lab studies of the CLL leukaemia cell line L1210 tend to show the promotional effects of CNCbl [110, 111, 112], or CNCbl lack of effect [113], and, by contrast, the cytotoxic effect of HOCbl [113, 114, 115], AdoCbl, and MeCbl [113] on the same L1210 CLL cell line.

It can be objected that, since all cobalamins are deconstructed on cell entry only to be reassembled in particular ratios of the varying forms [116], why should one form be more effective than another?

One answer is that giving supraphysiological doses of cobalamins may overwhelm this conversion, and even if it does not, high doses of the two active cobalamins, MeCbl and AdoCbl, on dealkylation, will supply both extra methyl groups, and adenine, which may be material to their individual mechanism of anticancer action. We have already seen that in one cancer model at least AdoCbl is significantly superior to MeCbl [78].

**FIGURE 6:**
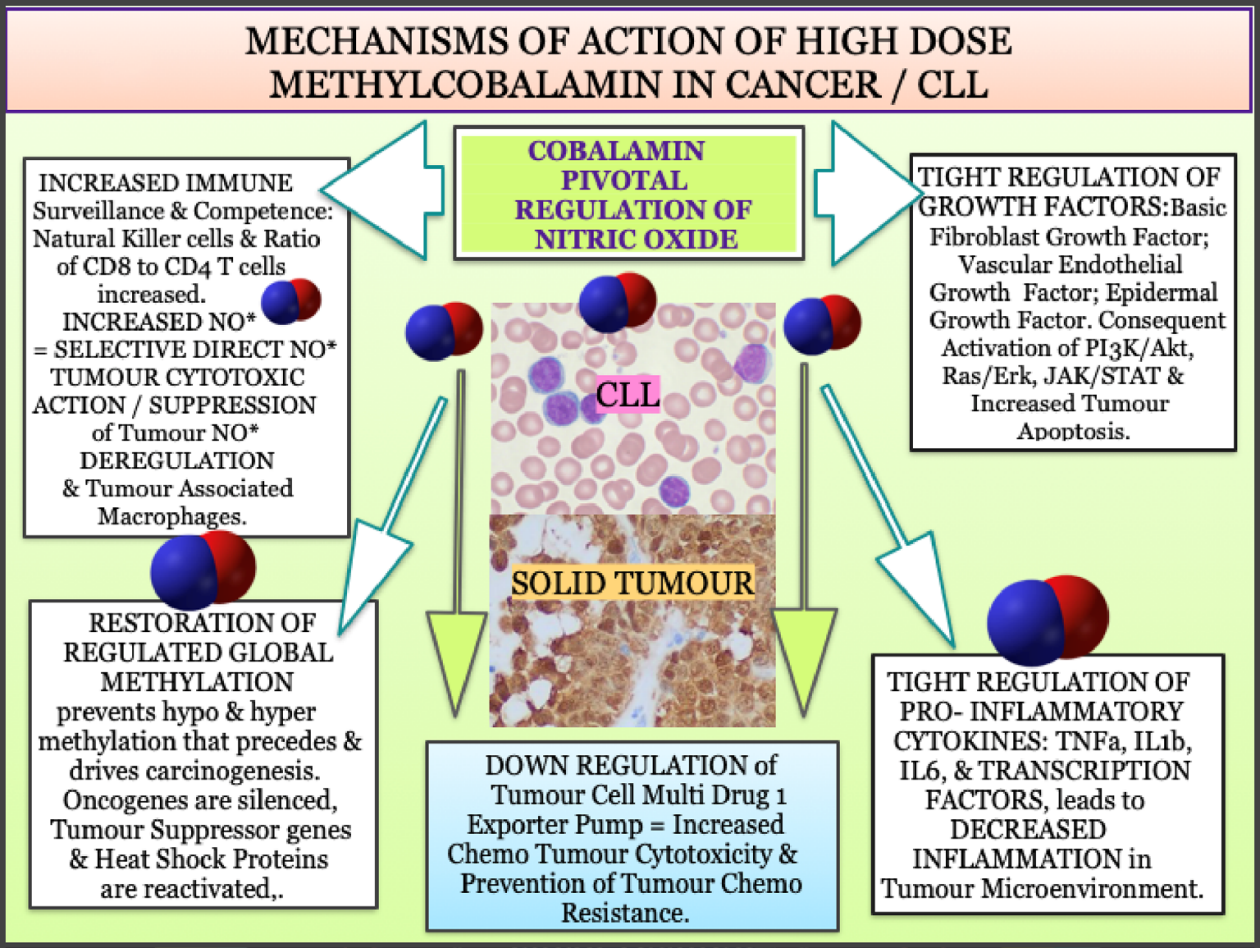
Mechanism of action of high dose methylcobalamin in cancer/CLL.

With respect to the potential impact of high dose MeCbl on CLL there are several known, general cobalamin anticancer mechanisms that may explain our patient's response: Figure 6.

First, the observed immune depression in CLL [117], will be countered by the known regulatory effects of cobalamin on the immune system. As in most cancers, natural killer (NK) cells are depressed in CLL [118], and the normal high ratio of CD8+ to CD4+ T cells is inverted with a consequent decreased immune tumour surveillance and cytotoxicity [119]. Conversely, cobalamin is known to increase levels of NK cells and raise the ratio of CD8+ to CD4+ T cells [120, 121, 122, 123]. Furthermore, there is in vitro work that shows that, of all intracellular cobalamins, MeCbl is the most potent immune regulator [124, 125]. This has been confirmed not only in B12 deficient patients, but also in healthy controls, with normal lymphocyte levels and CD8+ T cell counts increasing after 2 weeks of parenteral MeCbl treatment [122]. The very significant shift in our patient's CLL response with the change of treatment‐ and dose‐ from HOCbl to MeCbl (Figures 3 and 5), which was the cobalamin that actually cured his CLL, should be viewed in this context. Thus, with key immune defence players now fully armed, a chance infection could become the catalyst that alerted the immune response to finally eradicate the mutant CLL cells.

The in vitro cytotoxicity cobalamin cancer studies of Nishizawa's group indicate that a much higher dose would be needed in vivo, as the Cbl doses effective in Nishizawa's 1995 study (Figure 4) would translate into 5 g doses in an adult human. However, this would require IV administration, which would be both less practical, justifiable and more expensive in early CLL. The dose chosen for the patient was instead based both on the safety data, which shows that it is totally safe to inject 50–60 mg of MeCbl daily for 6 months [79], on the consistent effectiveness of this relatively low high dose in other serious pathologies, such as multiple sclerosis and motor neurone disease [79, 126], and on comparably effective doses in animal studies of sepsis [93, 94], and neuropathy [127]. The Watanabe et al. methylcobalamin for neuropathy rodent study is a particularly seminal paper, as it showed that for therapeutic purposes cobalamin dose really does matter, with a relatively low high dose being totally ineffective, and a much higher dose reversing neuropathy [127]. Even so, Watanabe's effective high dose for neuropathy in vivo is nowhere near as high as those in Nishizawa's in vitro cancer studies. It seems likely that this variation in high dose need may hold true for most pathologies in vivo, including different cancers, each one having its own threshold needs, or ID50.

Thus, by following the dose consensus of various research models, the minimal tumour cytoxic dose in vivo for this CLL patient was determined. It is, of course, quite possible that the addition of an immune stimulant such as BCG, or heat‐killed *Mycobacterium vaccae* and *Mycobacterium obuense* [128], to the high dose MeCbl protocol, to mimic the immune catalytic effect of an acute infection in our patient, may precipitate earlier and faster cures in CLL, and in particular in cases of CLL with poor prognostic genetics at diagnosis. It is also possible that, had an even larger dose been used from the start, (such as the 150 mg a day dose used during the severe UTI infection), eradication of the CLL clone could have been achieved sooner. All this remains to be determined in a planned pilot clinical study.

Second, there is now a body of work, much from the group of the neurologist Giuseppe Scalabrino in Milan, as from Japanese researchers, demonstrating that cobalamin is a central regulator of diverse growth factors (reviewed in [95]). Three of these are particularly pertinent here (two of them coincidentally over expressed in our patient's CLL cells, as the RGCC lab studies revealed). Levels of basic FGF are increasingly elevated in CLL progression [129], preventing apotosis of the clonal cells [130], while cobalamin, specifically as MeCbl, has been shown to lower FGF, and also lead to apoptosis, including in other FGF sensitive cancer cell lines [131]. Levels of VEGF are also key for CLL progression [132], whereas, again, cobalamin has been shown to be regulatory for VEGF [133, 134, 135, 136]. Levels of cobalamin are also directly correlated with EGF levels [137, 138], which in CLL progression are increasingly low and are an independent negative prognostic factor [139]. EGF is key for cell development, differentiation, growth and apoptosis through activation of signal transduction pathways such as PI3K/Akt, RAS/Erk, and JAK/STAT, which can both lead to and prevent apotosis. At least in neurological models, MeCbl is known to have regulatory effects for normal growth in the first two of these pathways [140, 141, 142]. So it may be reasonable to assume that, in CLL, MeCbl regulates levels of EGF back to normal, and thus will also work along these signalling pathways to increase apoptosis of CLL clonal cells. Indeed, it is also known that Cbl deficiency impacts negatively on apotosis [143, 144], so that conversely an abundance of Cbl may facilitate apoptosis as needed.

The question then remains: how does cobalamin regulate growth factor levels?

The same question can be asked of cobalamin's tight regulation of the immune system. Neither of its two classical mammalian coenzyme roles offers an obvious explanation. But we do know that cobalamin can regulate the universal second messenger nitric oxide/NO*, as opposed to just acting as an NO* mop, with downstream regulatory effects on cytokines and transcription factors [94], and the possibility that cobalamin, as AdoCbl, has a third mammalian coenzyme function as the primary nitric oxide synthase catalyst [145] may be relevant. Coincidentally, NO* is also a known regulator of growth factors, with potential to inhibit [146] and regulate FGF [146], has reciprocal regulation of VEGF [147, 148], as well as regulation of EGF and its receptor [149, 150], much depending on the levels of NO* generated [151].

The work of Altaie has already shown that cobalamin upregulates heat shock proteins concurrent with its upregulation of NO* [47]. Other research shows that NO* also upregulates heat shock proteins and sirtuins [152, 153]. SIRT1 is essential for expression of PGC1α, and both are known, among other signal pathways, also to be under the control of NO* [154, 155], and coincidentally, of cobalamin [156, 157, 158, 159, 160].

Deregulation of NO* itself is essential for oncogenesis and cancer progression [161]. Cobalamin pathways are also clearly deregulated both prior to, and as cancer progresses. I would propose that these two deregulations are intertwined and aetiological to oncogenesis and the progression of cancer, so that treatment with high dose parenteral cobalamin might reverse many of the deregulated NO* consequent immune deregulations, but also, via regulated NO* promotion, be directly tumour cytotoxic, with good outcomes. More work remains to be done in investigating this possibility. Be that as it may, high dose, parenteral MeCbl, some of which will be converted intracellularly to high levels of (even more tumour cytotoxic) AdoCbl [116], may one day prove to be a safe, economic and effective treatment for early stage CLL, and even perhaps for those CLL patients (40%) with negative cytogenetics at diagnosis, who with progression typically respond poorly to existing treatments.

## 10 Conclusion

There is no known curative treatment for CLL, which has an extremely low incidence of true so called ‘spontaneous’ remissions (0.6%). There is also no standard treatment for early stage CLL. Most oncologists still advocate a watch and wait strategy, as CLL can take many years to progress. However, the opportunity to treat his CLL with a research protocol that has almost no side effects, high dose parenteral methylcobalamin, when combined with the immune stimulation of a severe infection, resulted in the eradication of any CLL clonal disease, rather in the manner of the original immunotherapeutic cancer treatment, ‘Coley's toxins’.

The case also vividly illustrates the importance of using the right form, route and dose of cobalamins in CLL, and, indeed, in cancer in general.

The patient only made real progress towards complete immunophenotypic reversal of his CLL once the dose and form of cobalamin were changed, from parenteral low dose HOCbl to high dose MeCbl.

The review provided in Section 9 announces the important discovery—based on analysis of the entire field of cobalamin cancer studies in over 70 years, —that while the artefactual, commercial form of cobalamin, CNCbl, used for more than half a century, can be actively cancer promotional, and should *not* be used medically, the two active forms, AdoCbl and MeCbl have powerful anti‐cancer potential.

It is proposed that there is a need for clinical trials in CLL using high dose MeCbl, combined with an immune stimulant, such as heat killed mycobacteria/attenuated BCG vaccine. As the mildest of the leukaemias, it is possible that the model of treatment presented in this case report may lead to a radical change in prognosis, possibly even cures, for early and late CLL patients. This same protocol merits a trial in other haematological malignancies that still have no cure, including myelodysplastic syndrome and acute myeloid leukaemia.

## Ethics Statement

This case history has been written with the full knowledge and consent of the patient in question. The methylcobalamin, research‐based, experimental protocol was designed by the author, and prescribed privately, on compassionate grounds, by collaborating physicians, after the patient had given informed consent to treatment, which was self‐administered.

## Conflicts of Interest

The author declares no conflicts of interest.

## Supporting information


**Data S1.** Supporting Information.

## Data Availability

The data that support the findings of this study are available on request from the corresponding author. The data are not publicly available due to privacy or ethical restrictions.
