# Supplementary material for: Complete Immunophenotypic Reversal of Chronic Lymphocytic Leukaemia With High Dose Parenteral Methylcobalamin: A Case Report and Brief Review of Cobalamin in Cancer
Source: Cancer Rep (Hoboken). 2025 May 8;8(5):e70106. doi: 10.1002/cnr2.70106 (PMC12062518; doi:10.1002/cnr2.70106)
Supplement: Supplementary file 1 — Data S1. Supporting Information. [file CNR2-8-e70106-s001.pdf]

RESEARCH GENETIC CANCER CENTRE

R.G.C.C.-RESEARCH GENETIC CANCER CENTRE

Florina , 22 / 02 / 2008

Dear [redacted]

we send you the results from the analysis made about a patient [redacted] suffering from chronic lymphoblastic leukaemia stage I . The sample that was sent to us for analysis was a sample of 25ml of whole blood that contained EDTA-Ca as anti-coangulant , all packed with water ice .

In our laboratory we made the following :

- We isolated the malignant cells using Oncoquick with a membrane that isolates malignant cells from normal cells after centrifugation and positive selection using anti-CD19, anti-CD10, anti-CD138 and anti-CD38 cell marker, and negative selection using anti-CD45 particles (isolated 11,3cells/ml SD +/- 0,3cell).
- Then we developed cell cultures in a fetal calf serum media and at the same time we developed colony cultures in soft agar. In each culture of the well plate we added a chemotherapeutic substance that is used in clinical application. Then we developed those cultures and we harvested a sample every 24 hours for 6 days and made the following assays.
- There was made an isolation of the genomic DNA using the kit Invisorb of INVITEK .
- We isolated mRNA using the mRNA Magprep blood isolation kit of NOVAGEN.
- We traced the mRNA and the genes of MDR1 (multi drug resistant 1), MRP and LRP using the technique of Northern Blot .(resistance in drugs used in chemotherapies)
- We tracked the mRNA and the gene of topoisomerase I and II a & b using the technique of Northern Blot . (sensitivity in cytostatic inhibitors of topoisomerase)
- We tracked the quantity of the mRNA of the tubulin using the RT-PCR.(sensitivity in cytostatics of the kind of taxanes and the products of the alkaloids of Vinca)
- We defined the activity of the enzyme complex of the gloutathion-S- transferases (GST kit of NOVAgen) . (resistance in drugs used in chemotherapies- especially in platinum compounds)
- We defined the DNA methyl transferase which is a target of the alkyliating factors (products of platinum , cyclophosphamide and the products of it)
- We defined the mRNA of the thymidylate synthetase (TS) and the DHFR . (sensitivity in 5-FU, capecitabine and methotrexate)
- We defined the mRNA of the reductase of 5-CMP (sensitivity in gemcitabin)
- We defined the receptors of the MMP and the receptors of laminin (invasive ability of the tumor)
- We defined the expression of protein p27 that is responsible for cell arrest in G0 stage.
- We defined the VEGF (neoangiogenetic factor) and the induction of the apoptotic pathway using ONCOGENE kit from NOVAgen.
- We defined the ability of acting of the nucleous protein kinases which are a target of the carbazin compounds .
- We defined the overexpression of TGFa and TGFb factors as targets for suramin sulfate.

- We defined the overexression of somatostatin receptor (SS-R), of COX-2 and 5-LOX , of c-erb-B2 (Her/Neu2) , c-erb-B1, and androgen estrogen and progesterone receptors.

The above conclusions were also confirmed by the cell cultures of the tumor and in

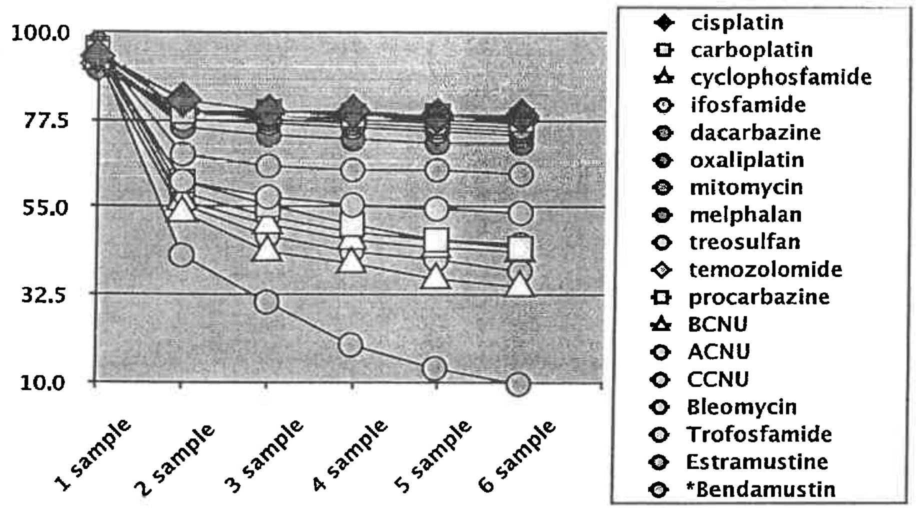

the diagrams there is a development curve for each category of cytostatics.

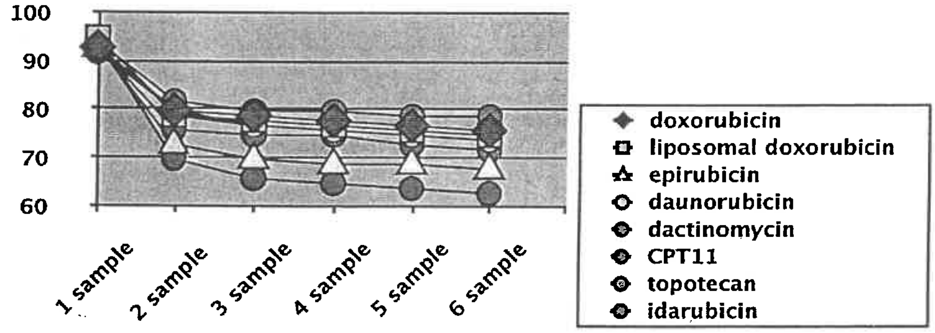

[redacted]

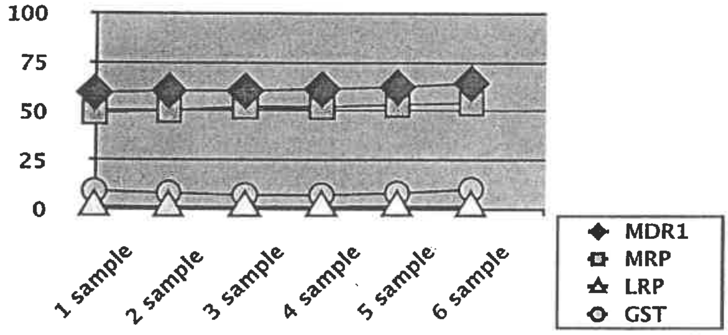

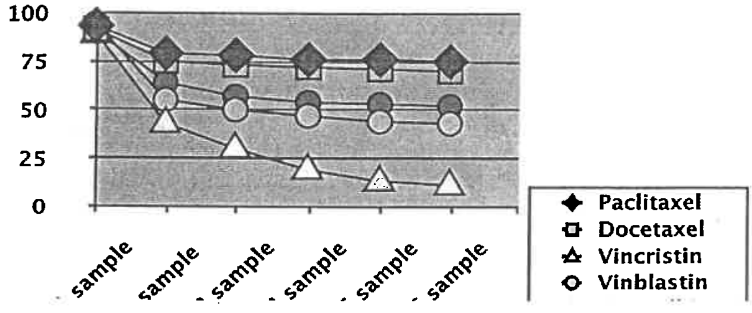

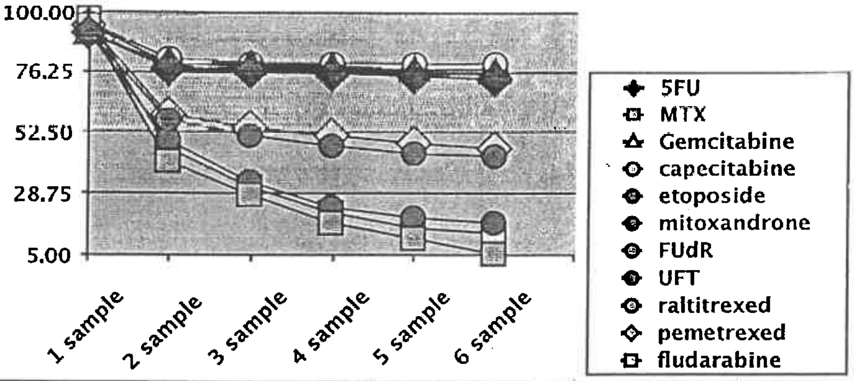

NAME	RELATED	RESULTS
CES1 &2 (carboxyesterase)	Resist to camptothecin	Normal
E2F1	Transcr. Fact of TS & topoI	Normal
p180	Tyrosin kinase growth f.	Normal
p27	Cell arrest (G_0)	55% over control
DPD	Resist to 5FU	Normal
UP	Resist to 5FU	Normal
NP	Resist to pyrim. antagonist	Normal
TP	Resist to 5FU	Normal
Gamma GC	Resist to alkyliating drug	Normal
p53	Cell cycle regulator	50% over control
p16	Apoptosis	45% over control
VEGF	Angiogenesis	40% over control
FGF	Angiogenesis	35% over control
PDGF	Angiogenesis	30% over control
COX2	Tumour Growth	Normal
5-LOX	Tumour Growth	Normal
MMP	Metastases	55% over control
TS	Rapid cell cycle (THFA)	Normal
DHFR	Rapid cell cycle (THFA)	Normal
SHMT	Rapid cell cycle (THFA)	Normal
GARFT	Rapid cell cycle(THFA)	Normal
NFκB	Transcription fact	35% over control
IκB (a,d,e)	Inhibitor of NFκB	30% below control
Ribonucleoside reductase	DNA synthesis	Normal
DNA methyltransferase I	DNA methylation	Normal
DNA demethylase	DNA methylation	Normal
O6-methylguanin-DNA-tran.	DNA methylation	Normal
TGF-b	Tumour Growth	30% over control
EGF	Tumour Growth	Normal
IGF	Tumour Growth	Normal
CypB1	Xenobiotic metabolism	Normal
CD20	Specific cell membrane ant.	Normal
Histone deacylase - dispeptide	DNA coiling(nucleosome)	Normal
c-erb-B2	Her/neu2	Normal

c-erb-B1	Her1	Normal
Bcr-abl	Resist phenotype	Normal
h-TERT (Human telomerase)	M2 crisis-aggresive phen.	10% over control

From the investigation above we concluded to the following :

1. From the whole neoplasmic population we have an expression of MDR1 in a percentage of 65% over control sample .(positive in the check of resistance)
2. The activity of GST is stable in the low limits (no resistance to platinum compounds)
3. The activity of gammaGC is stable in the low limits (no resistance to platinum compounds)
4. The activity of CES1 and CES2 is normal range (no resistance to camptothecin compounds)
5. The concentration of p180 is in normal range
6. Increased activity of the laminin and the MMP (increased invasive ability)
7. There is no sensitivity in taxanes (paclitaxel, docetaxel) and great sensitivity noticed in alkaloids of Vinca (especially in vincristin).
8. Minimal sensitivity noticed in 5FC, in 5-FU, in UFT , in FUdR in capecitabine, in raltitrexed, in pemetrexed and in gemcitabine but there is great sensitivity in methotrexate and in fludarabine.
9. Increased sensitivity in alkyliating factors (especially in bendamustin).
10. There is great overexpression of TGF b (30% over control) , of NFkBeta (35% over control) and EGF-r (<5%) growth factors and supression of expression of isoforms of IκB (a, d, e) (30% below control) .
11. It appears to have great sensitivity in the inhibitors of topoisomerase II a and II b (especially in mitoxantrone).
12. There is no sensitivity in the inhibitors of topoisomerase I .
13. There is no overexpression of SS-r receptor and there is no overexpression of 5-LOX mRNA, of estrogen receptor mRNA, of progesterone receptor mRNA, of COX-2 mRNA, c-erb-B2 ,of dehydrotestosterone receptor mRNA and of c-erb-B1.
14. We notice great neoangiogenetic ability (overexpression of VEGF-R – 40% over control sample).
15. Finally , there is no sensitivity in dacarbazine .
16. We notice that taurolidin cannot induce the apoptosis to the malignant cells (in IV route dosage).
17. We notice that taurolidin cannot induce the apoptosis to the malignant cells (in intraperitoneal route dosage)
18. We notice down-regulation of HSP 27 (Heat schock proteins) 27 (by 65% below control) , HSP 90 (20% below control) and HSP 72 (15% below control).

Conclusion :

- The specific tumor appears to have resisting populations because of the MDR1 overexpression that can be reversed by the use of verapamil combined with imidazole compounds (ketoconazole).
- The neoplasmatic cells have the greatest sensitivity in the alkyliating agent **bendamustin**, in the inhibitor of topoisomerase II **mitoxantrone** , in the antagonist **methotrexate** and **fludarabine** and in the inhibitors of tubulin dimmer polymerisation **vincristin**.
- **Also you can use:** bortezomib as inhibitor of proteasome over-activity and indirectly the transcriptional activity of NFκB, and bevacizumab as inhibitor of angiogenesis.

Regardly,

Dr Papasotiriou Ioannis MD
Head of molecular medicine dpt of
R.G.C.C.-RESEARCH GENETIC CANCER CENTRE

INDEX: M0 : Abnormal p16 , normal p53 and hTERT ,
M1: Normal hTERT , abnormal p53 , p16 ,
M2 crisis : over-expression of hTERT , p53 , p16
6th Sample viability : <20% greater sensitivity , 65%-20% partial sensitivity , >65% no sensitivity

[redacted]

ADDRESS : Florina-GR P.O. 53070
TEL : +30-24630-42264 , FAX: +30-24630-42265 Web site : www.rgcc-genlab.com E-mail :jpapasot@doctors.org.uk

RESEARCH GENETIC CANCER CENTRE

R.G.C.C.-RESEARCH GENETIC CANCER CENTRE

Florina , 22 / 02 / 2008

Dear [redacted]

we send you the results from the analysis made about a patient [redacted] suffering from chronic lymphoblastic leukemia stage I. The sample that was sent to us for analysis was a sample of 25ml of tissue sample that contained EDTA-Ca as anti-coangulant , all packed with water ice .

In our laboratory we made the following :

- We isolated the malignant cells using Oncoquick with a membrane that isolates malignant cells from normal cells after centrifugation and positive selection using anti-CD19, anti-CD10, anti-CD138 and anti-CD38 cell marker, and negative selection using anti-CD45 particles
- We develop two (2) different cultures from malignant cells (one with combination of vitamin c and vitamin K3 in ratio 100:1 and one other with the same substances in medium but in ration 50:1) .
- From each culture we test the expression of caspase 3 and the concentration of cytochrome c in the exctracellular matter (measurement of apoptosis induction)

The results are presented below :

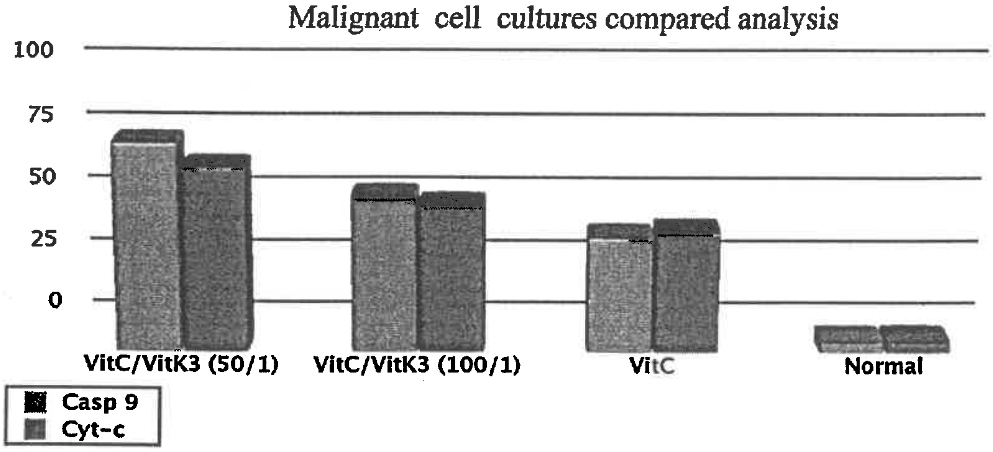

Conclusion : We notice that the combination in ration 50:1 can induce apoptosis for the specific cancer cells becoming from patient above better than the combination 100:1 .

Regardly,

Dr Papasotiriou Ioannis MD
Head of molecular medicine dpt of
R.G.C.C.-RESEARCH GENETIC CANCER CENTRE

[redacted]
ADDRESS : Florina-GR P.O. 53070
TEL : +30-24630-42264 , FAX: +30-24630-42265 Web site : www.rgcc-genlab.com E-mail ;jpapasot@doctors.org.uk

RESEARCH GENETIC CANCER CENTRE

R.G.C.C.-RESEARCH GENETIC CANCER CENTRE

Florina , 22 / 02 / 2008

Dear [redacted]

we send you the results from the analysis made about a patient [redacted] suffering from chronic lymphoblastic leukemia stage I. The sample that was sent to us for analysis was a sample of 25ml of whole blood that contained EDTA-Ca as anti-coangulant, all packed with water ice .

In our laboratory we made the following :

- We isolated the malignant cells using Oncoquick with a membrane that isolates malignant cells from normal cells after centrifugation and positive selection using anti-CD19, anti-CD10, anti-CD138 and anti-CD38 cell marker, and negative selection using anti-CD45 particles.
- We develop two (2) different cultures from malignant cells (one with vitD3 in the culture media –in concentration AUC- and one without vitD3) from the blood sample of patient above.
- From both cultures we measure the activity of caspase 3 , caspase 9 and cytochrome c .

The results are presented below :

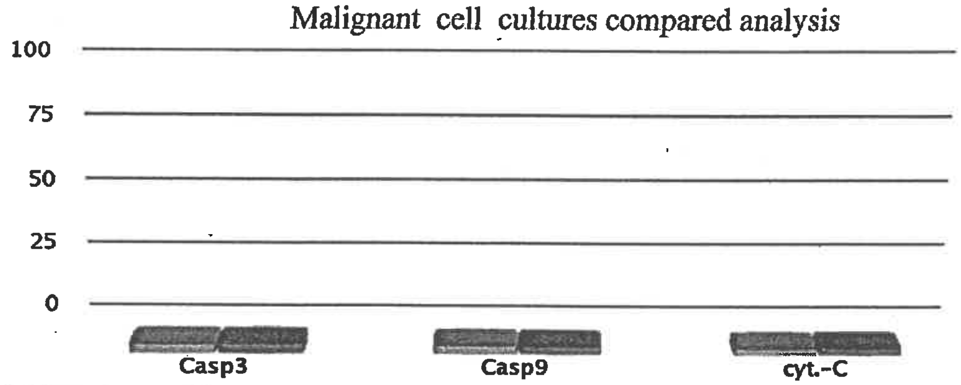

In the culture with vitamin D3 we notice no increase of caspase 3 activity and cytochrome c in compare with normal cell culture

Conclusion : We notice that vitamin D3 cannot induce the apoptosis to the cancer cell becoming from the patient above.

Regardly,

Dr Papasotiriou Ioannis MD
Head of molecular medicine dpt of
R.G.C.C.-RESEARCH GENETIC CANCER CENTRE

ADDRESS : Florina-GR P.O. 53070 [redacted]

TEL : +30-24630-42264 , FAX: +30-24630-42265 Web site : www.rgcc-genlab.com E-mail :jpapasot@doctors.org.uk

RESEARCH GENETIC CANCER CENTRE

R.G.C.C.-RESEARCH GENETIC CANCER CENTRE

Florina , 22 / 02 / 2008

Dear [redacted]

we send you the results from the analysis made about a patient [redacted] suffering from chronic lymphoblastic leukemia stage I. The sample that was sent to us for analysis was a sample of 15ml of whole blood that contained EDTA-Ca as anti-coangulant , all packed with water ice .

In our laboratory we made the following :

- We isolated the malignant cells using Oncoquick with a membrane that isolates malignant cells from normal cells after centrifugation and positive using anti-CD19, anti-CD20 and anti-CD38 and negative selection using anti-CD45 particles.
- We develop two (2) different cultures from malignant cells (one with thalidomide[th+] in the culture media –in concentration AUC- and one without thalidomide[th-]) from the blood sample of patient above.
- From the culture that include thalidomide [th+] to the media in the culture with malignant cells, we measure the activity of caspase 3 and cytochrome c .
- From both cultures we make compared analysis of VEGF , PDGF, FGF and MMPs inhibition rate.

The results are presented below :

Malignant cell cultures compared analysis

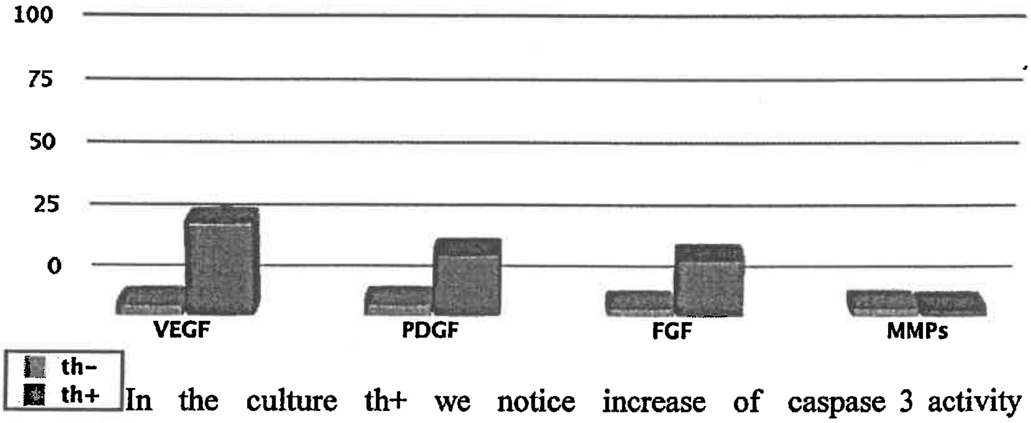

In the culture th+ we notice increase of caspase 3 activity and cytochrome c by 40% (apoptosis induction)

Conclusion : We notice that the thalidomide can inhibit the neovascularization and it can induce the apoptosis to the cancer cell becoming from the patient above, but it cannot inhibit the invasion activity of the cancer cells.

Regardly,

Dr Papasotiriou Ioannis MD

Head of molecular medicine dpt of

R.G.C.C.-RESEARCH GENETIC CANCER CENTRE

ADDRESS : Florina-GR P.O. 53070 [redacted]
TEL : +30-24630-42264 , FAX: +30-24630-42265 Web site : www.rgcc-genlab.com E-mail :jpapasot@doctors.org.uk

RESEARCH GENETIC CANCER CENTRE

R.G.C.C.-RESEARCH GENETIC CANCER CENTRE

Florina , 22 / 02 / 2008

Dear [redacted]

we send you the results from the analysis made about a patient [redacted] suffering from chronic lymphoid leukemia stage I. The sample that was sent to us for analysis was a sample of 25ml of whole blood that contained EDTA-Ca as anti-coangulant , packed with water ice .

In our laboratory we made the following :

- We isolated the malignant cells using Oncoquick with a membrane that isolates malignant cells from normal cells . Then we centrifuged at 350g for 10 min and we collected the supernatant with the malignant cells . Then we proceed to isolation of malignant cells from mononuclear cells by negative selection .
- Then we developed thirty nine cell cultures in a fetal calf serum media . In each culture of the well plate we added a biological modifier substance (H2O2, ascorbic acid , carnivora , misteltoe, quercetin , indol-3-carbinol , c-statin , ukrain , polyMVA, co enzyme Q10, essiac tea, modified citrus pectin, IP6 , pacreatic enzymes, salvestrol, Uncaria Tomentosa, carctol, noni juice, annonaceous acetogenins, caesium chloride, reolysin, amygdalin-B17-, artesunate, maitake, lycopene, curcumin, green tee extract, melatonin, ellagic acid, L-methionine, N-acetyl-cystein, Niacin (Vit.B3), L-carnithine, Vitamin E (tocopherol), superoxide dismutase (SOD), propolis, selenium, aloe vera, IFNa2) that is used in clinical application. Then we developed those cultures and we harvested a sample every 24 hours and made the following assays.
- In the culture that it contains all substance we measure the apoptotic ability using the oncogen apoptosis kit
- In the culture that it contains the carnivora we measure the inhibition of tyrosin kinase catalytic ability from growth factors receptor (EGF-r, IGF-r,) and the production of cytokines.
- In the culture that it contains the Ukrain we measure the inhibition of tyrosin kinase catalytic ability from growth factors receptor (EGF-r, IGF-r,) and the production of cytokines PMBC
- In the culture that contains quercetin we measure the inhibition of EGF and IGF .
- In the culture that contains indol-3-carbinol we measure the inhibition of VEGF and FGF and PDGF
- In the culture that it contains the misteltoe we measure the inhibition of tyrosin kinase catalytic ability from growth factors receptor (EGF-r, IGF-r,) and the production of cytokines and the increase of PMBC
- In the culture that it contains the H2O2 we measure viability of the culture in 4 days of treatment.
- In the culture that it contains the ascorbic acid we measure the catalytic activity of GSH and GSSG (redox reaction) and the induction of cytochrome C (apoptosis).

- In the culture that it contains the PolyMVA we measure the catalytic activity of GSH and GSSG (redox reaction) and the induction of cytochrome C (apoptosis)
- In the culture that it contains the artesunate we measure the catalytic activity of GSH and GSSG (redox reaction for free radical since artesunate bind free radicals with iron molecule) , the inhibition of VEGF , FGF and PDGF (since it act to the angiogenesis cascade reactions) and the induction of cytochrome C (apoptosis).

RESULTS:

1. We notice that in culture that contains the ascorbic acid we have increase of the cascade of caspase (especially 3 and 9) and cytochrom-c by 55%.
2. We notice that in culture that contains the PolyMVA we have no increase of the cascade of caspase (especially 3 and 9) and cytochrom-c.
3. We notice that in culture that contains carnivora we have inhibition of EGF-r by less than 5% and for IGF-r by 5% and we notice no increase of cytokine production 5% .
4. We notice that in the culture that contains quercetin we have inhibition of EGF by 60% and IGF by 45%
5. We notice that in the culture that contains indol-3-carbinol we have inhibition of VEGF by less than 5% , of FGF by 5% , and PDGF by 5%
6. We notice that in culture that contains misteltoe we have inhibition of EGF-r by less than 5% and for IGF-r by 5% and we notice no increase of cytokine production , and there is no increase of PMBC .
7. We notice that in culture that contains the c-statin we have increase of the cascade of caspase (especially 3 and 9) and cytochrom-c by 55%.
8. We notice that in culture that contains Ukrain we have inhibition of EGF-r by less 5% and for IGF-r by 5% and we notice no increase of cytokine production , and there is no increase of PMBC.
9. We notice that in culture that contains the H2O2 we have increase of the cascade of caspase (especially 3 and 9) and cytochrom-c by less than 5% and the viability of the culture remain stable .
10. We notice that in culture that contains the co enzyme Q10 we have increase of the cascade of caspase (especially 3 and 9) and cytochrom-c by less than 5% and the viability of the culture remain stable .
11. We notice that in culture that contains the essiac tea we have increase of the cascade of caspase (especially 3 and 9) and cytochrom-c by less than 5% and the viability of the culture remain stable .
12. We notice that in culture that contains the modified citrus pectin we have increase of the cascade of caspase (especially 3 and 9) and cytochrom-c by less than 5% and the viability of the culture remain stable .
13. We notice that in culture that contains the IP6 we have increase of the cascade of caspase (especially 3 and 9) and cytochrom-c by less than 5% and the viability of the culture remain stable .
14. We notice that in culture that contains the pacreatic enzymes we have increase of the cascade of caspase (especially 3 and 9) and cytochrom-c by less than 5% and the viability of the culture remain stable .

15. We notice that in culture that contains the salvestrol we have increase of the cascade of caspase (especially 3 and 9) and cytochrom-c by 65% and the viability of the culture reduced by 45%.
16. We notice that in culture that contains the uncaria tomentosa we have increase of the cascade of caspase (especially 3 and 9) and cytochrom-c by less than 5% and the viability of the culture remain stable.
17. We notice that in culture that contains the caesium chloride we have no increase of the cascade of caspase (especially 3 and 9) and cytochrom-c and the viability of the culture remain stable.
18. We notice that in culture that contains the carctol we have increase of the cascade of caspase (especially 3 and 9) and cytochrom-c by less than 5% and the viability of the culture remain stable.
19. We notice that in culture that contains the noni juice we have increase of the cascade of caspase (especially 3 and 9) and cytochrom-c by less than 5% and the viability of the culture remain stable.
20. We notice that in culture that contains the annonaceous acetogenins we have increase of the cascade of caspase (especially 3 and 9) and cytochrom-c by less than 5% and the viability of the culture remain stable.
21. We notice that in culture that contains the reolysin we have increase of the cascade of caspase (especially 3 and 9) and cytochrom-c by less than 5% and the viability of the culture remain stable.
22. We notice that in culture that contains the amygdalin-B17- we have no increase of the cascade of caspase (especially 3 and 9) and cytochrom-c and the viability of the culture remain stable.
23. We notice that in culture that contains maitake we have inhibition of EGF-r by less than 5% and for IGF-r by 5% and we notice no increase of cytokine production , and there is no increase of PMBC.
24. We notice that in culture that contains the curcumin (turmeric) we have no increase of the cascade of caspase (especially 3 and 9) and cytochrom-c and the viability of the culture remain stable.
25. We notice that in culture that contains the lykopene we have no increase of the cascade of caspase (especially 3 and 9) and cytochrom-c and the viability of the culture remain stable.
26. We notice that in culture that contains the green tea extract we have no increase of the cascade of caspase (especially 3 and 9) and cytochrom-c and the viability of the culture remain stable
27. We notice that in culture that contains artesunate , there is inhibition of redox reaction and no increase of intracellular free radicals , there is increase of cytochrome c (apoptosis) by 45% and the inhibition rate of VEGF is 35% , of FGF is 30% and of PDGF is 25%.
28. We notice that in culture that contains the melatonin we have no increase of the cascade of caspase (especially 3 and 9) and cytochrom-c and the viability of the culture remain stable.
29. We notice that in culture that contains the ellagic acid we have no increase of the cascade of caspase (especially 3 and 9) and cytochrom-c and the viability of the culture remain stable.
30. We notice that in culture that contains the L-methionine we have no increase of the cascade of caspase (especially 3 and 9) and cytochrom-c and the viability of the culture remain stable.

31. We notice that in culture that contains the N-acetyl-cystein we have no increase of the cascade of caspase (especially 3 and 9) and cytochrom-c and the viability of the culture remain stable.
32. We notice that in culture that contains the niacin we have no increase of the cascade of caspase (especially 3 and 9) and cytochrom-c and the viability of the culture remain stable.
33. We notice that in culture that contains the L-carnithine we have no increase of the cascade of caspase (especially 3 and 9) and cytochrom-c and the viability of the culture remain stable.
34. We notice that in culture that contains the vitamin E we have no increase of the cascade of caspase (especially 3 and 9) and cytochrom-c and the viability of the culture remain stable.
35. We notice that in culture that contains the superoxide dismutase we have no increase of the cascade of caspase (especially 3 and 9) and cytochrom-c and the viability of the culture remain stable.
36. We notice that in culture that contains the aloe vera extract we have no increase of the cascade of caspase (especially 3 and 9) and cytochrom-c and the viability of the culture remain stable.
37. We notice that in culture that contains selenium we have inhibition of EGF-r by less than 5% and for IGF-r by 5% and we notice no increase of cytokine production, and there is no increase of PBMC and NK.
38. We notice that in culture that contains IFNa2 we have inhibition of EGF-r by less than 5% and for IGF-r by 5% and we notice no increase of cytokine production, and there is no increase of PMBC.
39. We notice that in culture that contains the propolis we have increase of the cascade of caspase (especially 3 and 9) and cytochrom-c by 40%.

CONCLUSION: It seems that this specific population of malignant cell have greater sensitivity in quercetin, in ascorbic acid, in artesunate, in propolis, in salvestrol and in c-statin and less in hydrogen peroxide (H_2O_2), co enzyme Q10, essiac tea, modified citrus pectin, IP6 , N-acetyl-cystein, pacreatic enzymes, caesium chloride, in carnivora, ellagic acid, in L-carnithine, in L-methionine, vitamin E (tocopherol), maitake, in IFNa2, in ukrain, in amygdalin-(B17), in superoxide dismutase, in curcumin (turmeric), in misteltoe, in Poly-MVA, in indol – 3-carbinol, uncaria tomentosa (samento), in melatonin, in selenium, carctol, noni juice, niacin, aloe vera extract, annonaceous acetogenins (paw paw), reolysin (reovirus), lykopene and in green tea extract.

Regardly,

Dr Papasotiriou Ioannis MD
Head of molecular medicine dpt of

[redacted]

ADDRESS : Florina-GR P.O. 53070
TEL : +30-24630-42264 , FAX: +30-24630-42265 Web site : www.rgcc-genlab.com E-mail :jpapasot@doctors.org.uk
